# Molecular Mechanisms of Grain Chalkiness Variation in Rice Panicles

**DOI:** 10.3390/plants14020244

**Published:** 2025-01-16

**Authors:** Zhong Li, Min Xi, Youzun Xu, Xueyuan Sun, Debao Tu, Yongjin Zhou, Yalan Ji, Linsheng Yang

**Affiliations:** Rice Research Institute, Anhui Academy of Agricultural Sciences, Hefei 230031, China; lizhong021@126.com (Z.L.); xuyouzun@aaas.org.cn (Y.X.); sunxueyuan2024@163.com (X.S.); tudebao@aaas.org.cn (D.T.); zhouyongjin1111@163.com (Y.Z.); jiyalan@aaas.org.cn (Y.J.); yangls20160601@163.com (L.Y.)

**Keywords:** rice (*Oryza sativa* L.), grain filling rate, grain chalkiness, gene expression, starch

## Abstract

Grain chalkiness adversely affects rice quality, and the positional variation of grain chalkiness within a rice panicle presents a substantial obstacle to quality improvement in China. However, the molecular mechanism underlying this variation is unclear. This study conducted a genetic and physiological analysis of grains situated at distinct positions (upper, middle, and bottom primary branches of the rice panicle, denoted as Y1, Y2, and Y3) within a rice panicle using the Yangdao 6 variety. The results indicated that the percentage of chalky grains (PCG) in Y1 was the highest, i.e., 17.12% and 52.18% higher than that of Y2 and Y3, respectively. Y2 exhibited the highest degree of grain chalkiness (DGC), attributable to its greater area of endosperm chalkiness (AEC) than the others. Y3 demonstrated the lowest PCG and DGC. Additionally, Y1 and Y2 were characterized by lower amylose and protein contents, as well as looser starch granule morphology, in comparison to Y3. Compared with Y3, both the average and maximum filling rates of Y1 and Y2 increased markedly; however, the active filling duration was notably reduced by 7.10 d and 5.56 d, respectively. The analysis of genomic expression levels indicated an enrichment of starch and sucrose metabolism in Y1-vs.-Y2, Y2-vs.-Y3, and Y1-vs.-Y3, with 7 genes (5 up-regulated and 2 down-regulated), 53 genes (12 up-regulated and 41 down-regulated), and 12 genes (2 up-regulated and 10 down-regulated) in the Y1-vs.-Y2, Y2-vs.-Y3, and Y1-vs.-Y3. The majority of these genes were down-regulated, linking metabolic activity to grain filling and contributing to the occurrence of grain chalkiness in rice panicles. In conclusion, the metabolic processes associated with sucrose and starch play a crucial role in regulating grain filling and the formation of chalkiness in rice.

## 1. Introduction

Rice (*Oryza sativa* L.) is a globally significant cereal crop, serving as a stable food for approximately 60% of the world’s population. In recent years, economic growth and improvements in quality of life have led to an increased consumer demand for high-quality rice. Consequently, enhancing rice quality has become a priority in China, second only to yield improvement. A critical factor limiting the production of high-quality rice is grain chalkiness, an undesirable trait that negatively impacts rice’s appearance, milling, and cooking properties, thereby reducing its market value [1,2]. For instance, the chalky tissue in rice exhibits loosely packed starch granules and protein bodies, resulting in lower viscosity but higher gelatinization enthalpy compared to translucent rice various [3,4]. Grain chalkiness frequently occurs in numerous rice-producing regions globally, particularly under conditions of climate-induced high temperatures [5]. Furthermore, the physical appearance quality of rice plays a crucial role in the approval of new varieties. The presence of quality defects due to grain chalkiness has impeded the selection of several high-yielding and abiotic stress-resistant rice varieties by breeders. Consequently, it is becoming increasingly crucial to understand the underlying mechanisms responsible for the occurrence of grain chalkiness.

Spikelet development within the rice panicle progresses in a basipetal [6]. The spatial arrangement of spikelets within the panicle significantly influences both grain dry weight and quality [7]. Previous studies have revealed that grain chalkiness differs across spikelet positions within the panicle. Specifically, chalky kernels were found to be least prevalent in the primary branches located in the upper region of the panicle, whereas they were most prevalent in the secondary branches of the lower panicle [8]. Kernels located on the upper primary branches of the rice panicle exhibited accelerated development and typically had greater grain weight, enhanced starch accumulation, and reduced chalkiness in comparison to those on the lower branches [9]. Multiple studies have corroborated that chalky kernels are more prevalent on the top and primary rachis, even under conditions of high nitrogen fertilizer application to the rice crops [10,11]. Furthermore, grains on the upper primary branch of the rice panicle demonstrated higher levels of chalkiness than those on the lower secondary branch [10,12]. The observed variability in chalkiness among grains located at different spikelet positions within a rice panicle adversely affects grain quality, rendering them less desirable to consumers. The prevalent cultivation of rice varieties characterized by large panicles and high yield potential is anticipated to exacerbate this issue in China. Therefore, mitigating chalkiness in grains located at different positions within a rice panicle is a promising strategy for enhancing overall grain quality.

The formation of chalkiness in rice grains is a complex physiological process intricately linked to the grain filling phase. The metabolism of carbon- and nitrogen-containing compounds during grain filling is crucial for rice quality [13]. Previous research has indicated that a reduction in the activity of starch synthesis enzymes or an up-regulation of α-amylose gene expression within the endosperm may enhance the incidence of grain chalkiness, particularly under conditions of high-temperature stress during grain development [14,15]. An enhancement in the activity of enzymes associated with nitrogen metabolism-expedited protein accumulation and induced grain chalkiness in rice subjected to elevated nitrogen conditions [10,16]. However, research has been unable to comprehensively elucidate the variations in the chalkiness of rice grains originating from different spikelet positions solely based on the perspective of assimilate concentration within the grains [11]. A supported study by Yang et al. [17] demonstrated that carbohydrate supply was not the primary limiting factor for the development of spikelets in the lower branches of the panicle. Mohapatra et al. [9] indicated that the suboptimal grain quality observed in the basal sections of the panicle was not attributable to a deficiency in assimilates. Instead, it was due to diminished sink efficiency in the conversion of soluble assimilates into reserve structural compounds. In addition, numerous genes implicated in starch and protein synthesis, including OsPPDKB [18], OsAGPL4 [19], UGPase1 [20], PDIL1-1 [21], BiP [22], and *Chalk*5 [23], have been identified as contributors to the variation in rice grain chalkiness. However, prior research on rice chalkiness has predominantly concentrated on entire grains sourced from various or specific sections of the panicle within a given variety [13,24,25]. There are few studies on investigating the grain chalkiness at different spikelet positions in rice panicles.

The positional variation of grain chalkiness suggests that a complex network of potential regulatory pathways is involved in the formation of chalkiness in rice grains. Limited information is available on the molecular mechanisms underlying the occurrences of chalkiness in grains located at various positions within rice panicles. In this study, we employed rice varieties demonstrating notable positional variations in grain chalkiness as the experimental materials to perform high-throughput sequencing analysis on developing seeds located at various positions within a rice panicle. Additionally, we investigated the phenotypic and physicochemical properties of the mature rice seeds. The objectives of this study were to: (1) examine the variations in grain chalkiness and endosperm structures among kernel positions within a rice panicle, (2) analyze the grain filling characteristics associated with different positions within a panicle, and (3) elucidate the molecular mechanisms underlying the occurrence of chalkiness in grains located at different positions on the panicle using transcriptomic techniques.

## 2. Results

### 2.1. Chalkiness and Physicochemical Properties of Mature Grains

The chalky grains located at different spikelet positions within a rice panicle exhibited contrasting differences (Table 1). The percentage of chalky grains (PCG) of Y1 was the highest, with increases of 17.12% and 52.18% compared to Y2 and Y3, respectively. The area of endosperm chalkiness (AEC) of Y1 was significantly lower than that of Y2 and comparable to that of Y3. Consequently, due to the high AEC, the degree of grain chalkiness (DGC) in Y2 was significantly higher than that in Y1 and Y3. Y3 eventually exhibited the lowest PCG and DGC. Grain weight did not show a complete correlation with grain position. Among the grains attached to different positions within a rice panicle, those attached to Y3 had both higher amylose and protein contents, resulting in larger grain weight. Additionally, the grain length/width (L/B) ratio followed an order of Y3 > Y2 > Y1.

### 2.2. Micro-Scale Observations of the Endosperm Structure

**Figure 1:**
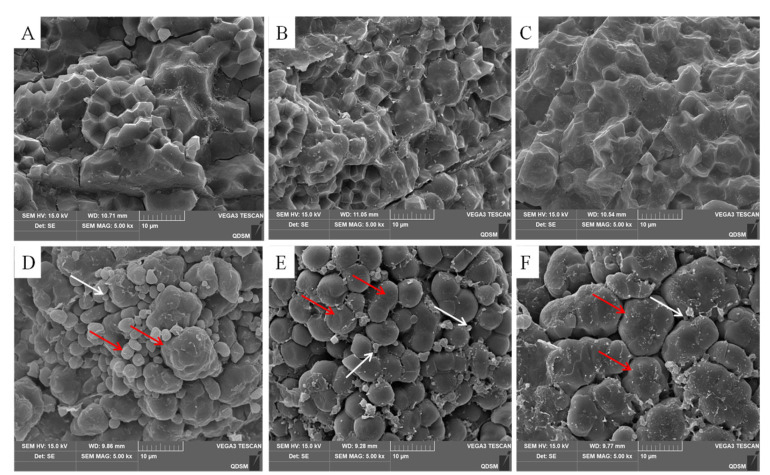
SEM images of the grain endosperm structure. (**A**–**C**) indicate endosperm structure of translucent grains of Y1, Y2, and Y3; D, E, and F indicate endosperm structure of the chalky grains of Y1, Y2, and Y3. Y1, the grains attached to the four top primary branches of the rice panicle; Y2, the grains attached to the middle primary branches of the rice panicle; Y3, the grains attached to the four bottom primary branches of the rice panicle. Red arrows point to the starch granules, and white arrows point to the protein bodies accumulated around the starch granules. (**D**–**F**) images are related to the parts of the ventral side covered with chalkiness.

SEM images revealed distinct positional differences in the distribution of endosperm starch granules within a rice panicle (Figure 1). Notably, the translucent endosperm exhibited densely packed polyhedral granules without any observed air spaces, irrespective of grain position. In contrast, chalky endosperms typically displayed loosely arranged starch granules with evident globular protein bodies occupying inter-granule spaces. Moreover, the heterogeneity of starch granules was a characteristic microstructure found in Y1, which contained irregular amyloplasts and small, simple spherical granules. Y2 predominantly consisted of numerous small elliptical or other spherical starch granules. Comparatively, Y3 exhibited slightly superior endosperm starch granularity compared to Y1 and Y2.

### 2.3. Grain Filling Characteristics of Rice Gain from Different Positions in a Panicle

Grain filling is an important physiological process that contributes greatly to grain quality and yield. In this study, differences in the grain-filling dynamics of the grains at different positions within a rice panicle were analyzed (Table 2). The GR_0_ value in Y3 was higher than those in Y1 and Y2. T_max_ showed a descending order of Y3, Y2, and Y1. Furthermore, both Y1 and Y2 displayed significantly elevated levels of GR_max_ and GR_mean_ when compared to Y3. Consequently, the D of Y3 significantly surpassed that of Y1 and Y2 by 7.10 d and 5.56 d, respectively. The GI of Y3 had the largest value, followed by Y2, and finally Y1.

### 2.4. DEGs in Grains from Different Spikelet Positions in a Rice Panicle

**Figure 2:**
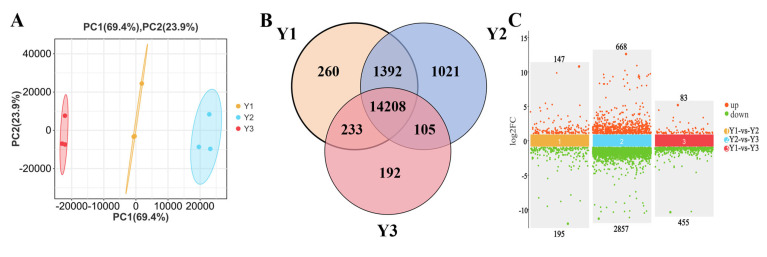
Principal component analysis (PCA) of differentially expressed genes in grains (**A**). Venn analysis of common and specific genes in different parts of the rice panicle (**B**). Differentially expressed genes between the Y1-vs.-Y2, Y2-vs.-Y3 and Y1-vs.-Y3 (**C**). Y1, Y2, and Y3 indicate the grains attached to the four top primary branches, the remaining middle primary branches, and the four bottom primary branches of the rice panicle. The RNA-Seq dataset comprises three biological replicates.

The variations in gene expression in developing rice grains at different positions within a panicle were analyzed using transcriptome technology in this study. Principal component analysis (PCA) demonstrated significant separation among Y1, Y2, and Y3, with good repeatability, indicating substantial differences among Y1, Y2, and Y3 (Figure 2A).

Transcriptome results showed that a total of 17,411 genes were identified in Y1, Y2, and Y3. Among them, 16,093, 16,724, and 14,738 were identified in Y1, Y2, and Y3 samples, respectively (Figure 2B). In addition, 14,208 genes were identified in Y1, Y2, and Y3; 1392, 233, and 105 genes were identified in Y1-vs.-V2, Y1-vs.-Y3, and Y2-vs.-Y3; and 260, 1021, and 192 genes were identified only in Y1, Y2, and Y3. In the Y1-vs.-Y2 comparison, a total of 342 DEGs were identified, with 147 up-regulated and 195 down-regulated. In the Y2-vs.-Y3 comparison, a total of 3525 DEGs were identified (688 up-regulated and 2857 down-regulated). Furthermore, in the Y1-vs.-Y3 comparison, there were 538 DEGs (83 up-regulated and 455 down-regulated) (Figure 2C).

### 2.5. Functional Classification of DEGs in Grains from Different Positions of the Panicle

**Figure 3:**
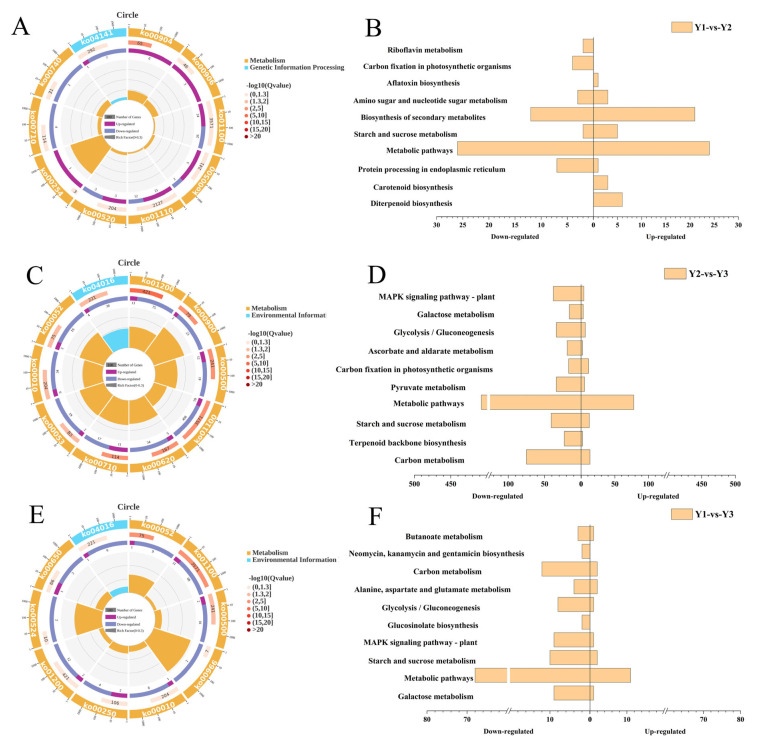
KEGG pathways enrichment analysis in Y1-vs.-Y2 (**A**), Y2-vs.-Y3 (**C**) and Y1-vs.-Y3 (**E**), and functional characterization between Y1-vs.-Y2 (**B**), Y2-vs.-Y3 (**D**) and Y1-vs.-Y3 (**F**). Y1, Y2, and Y3 indicate the grains attached to the four top primary branches, the remaining middle primary branches, and the four bottom primary branches of the rice panicle, respectively.

DEGs in Y1-vs.-Y2 were subjected to enrichment analysis. In the Y1-vs.-Y2 comparison, a total of 120 genes were identified (Figure 3A). Among them were up-regulated genes involved in diterpenoid biosynthesis (6 genes), carotenoid biosynthesis (3 genes), starch and sucrose metabolism (7 genes), and biosynthesis of secondary metabolites (24 genes), as well as a single up-regulated gene associated with aflatoxin biosynthesis. Conversely, protein processing in the endoplasmic reticulum exhibited one up-regulated gene, while seven others were down-regulated. Metabolic pathways showed a mixed pattern, with 24 up-regulated and 26 down-regulated genes (Figure 3B). Carbon fixation in photosynthetic organisms had four down-regulated genes, and finally, riboflavin metabolism displayed one down-regulated gene.

In the Y2-vs.-Y3 comparison, several metabolic pathways, including carbon metabolism, terpenoid backbone biosynthesis, starch and sucrose metabolism, metabolic pathways, pyruvate metabolism, carbon fixation in photosynthetic organisms, ascorbate and aldarate metabolism, and glycolysis/Ten metabolic pathways, including gluconeogenesis, galactose metabolism, and MAPK signaling pathway–plant, were significantly enriched and down-regulated (Figure 3C,D). A total of 841 genes were identified.

In the Y1-vs.-Y3 comparison, several metabolic pathways, including galactose metabolism, metabolic pathways, starch and sucrose metabolism, MAPK signaling pathway–plant, glucosinolate biosynthesis, glycolysis/gluconeogenesis, alanine, aspartate and glutamate metabolism, carbon metabolism, and neomycin, as well as ten metabolic pathways, including kanamycin and gentamicin biosynthesis and butanoate metabolism, were significantly enriched, and the expression of these pathways was down-regulated. A total of 148 genes were identified in these pathways (Figure 3E,F).

The above results demonstrated a significant down-regulation of the gene expression in Y3 when compared to Y1 and Y2, indicating slower grain filling in the bottom primary branches of the rice panicle. Further Venn analysis revealed that metabolic pathways (ko01100) and starch and sucrose metabolism (ko00500) were enriched in all three comparisons: Y1-vs.-Y2, Y2-vs.-Y3, and Y1-vs.-Y3. In the comparisons of Y1-vs.-Y2 and Y2-vs.-Y3, carbon fixation in photosynthetic organisms was exclusively co-enriched. Additionally, carbon metabolism, glycolysis/gluconeogenesis, galactose metabolism, and MAPK signaling pathway–plant were specifically co-enriched only in the comparisons of Y2-vs.-Y3 and Y1-vs.-Y3 (Table 3).

### 2.6. DEGs Related to Starch and Sucrose Metabolism

**Figure 4:**
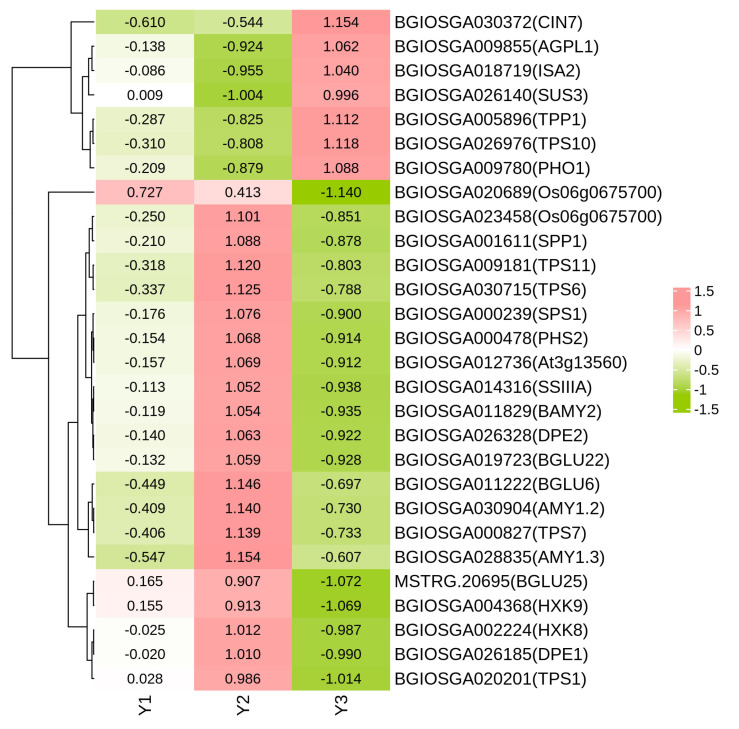
Expression of genes related to starch sucrose metabolism. Y1, Y2, and Y3 indicate the grains attached to the four top primary branches, the remaining middle primary branches, and the four bottom primary branches of the rice panicle, respectively. Y1, Y2, and Y3 each had 3 biological replicates.

**Figure 5:**
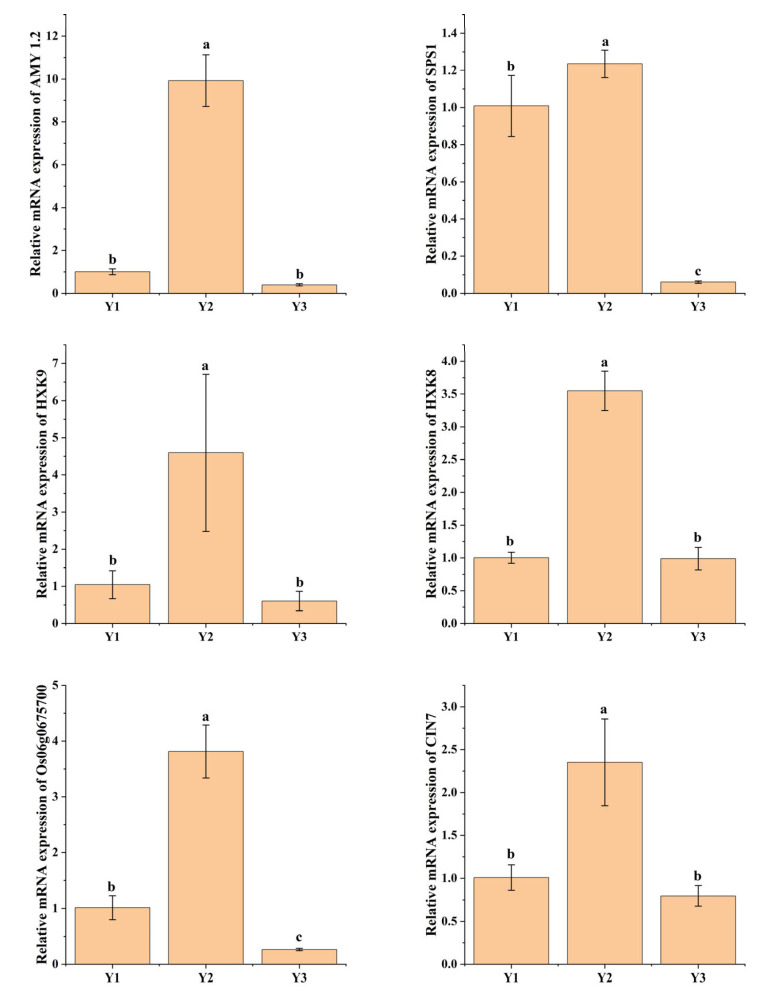
The qRT-PCR-verified expression of genes related to starch sucrose metabolism. Y1, Y2, and Y3 indicate the grains attached to the four top primary branches, the remaining middle primary branches, and the four bottom primary branches of the rice panicle. Different lowercase letters in the same column indicate significant differences at *p* < 0.05 (n = 3).

In the Y1-vs.-Y2 comparison, there were seven DEGs involved in starch and sucrose metabolism, with five up-regulated and two down-regulated genes. Similarly, in the Y2-vs.-Y3 comparison, a total of 53 DEGs were identified, including 12 up-regulated and 41 down-regulated genes. In the Y1-vs.-Y3 comparison, there were 12 DEGs related to starch and sucrose metabolism, consisting of 2 up-regulated and 10 down-regulated genes. Moreover, after eliminating duplicate occurrences of the same gene (which appeared multiple times), a total of 28 unique genes were found across all three treatments (Figure 4). These genes were identified to be related to starch and sucrose metabolism. Some of these genes are primarily involved in sucrose synthesis and degradation, such as BGIOSGA000239 (SPS1), BGIOSGA000239 (SPS1), BGIOSGA026140 (SUS3), BGIOSGA000478 (PHS2), and BGIOSGA001611 (SPP1). Some genes play key roles in the starch synthesis and degradation pathway, such as BGIOSGA009855 (AGPL1), BGIOSGA014316 (SSIIIA), BGIOSGA018719 (ISA2), BGIOSGA026185 (DPE1), BGIOSGA026328 (DPE2), BGIOSGA028835 (AMY1.3), and BGIOSGA030904 (AMY1.2). In this study, only one gene exhibited higher expression levels in Y1 compared to Y2 and Y3. Among the remaining 27 genes, 7 demonstrated the highest expression in Y3, while the expression of 20 genes peaked in Y2. Based on this premise, the present study employed qRT-PCR to validate the expression of *SPS1*, *HXK8*, *HXK9*, *Os06g0675700*, *CIN7,* and *AMY1.2*, which exhibited consistent change patterns with the transcriptome analysis (Figure 5). These findings suggest that genes associated with starch sucrose metabolism were up-regulated in Y1 and Y2 compared to Y3, being the highest in Y2, indicating a significantly weaker starch sucrose metabolism in Y3 compared to Y1 and Y2.

### 2.7. DEGs Related to Carbon Metabolism and Galactose Metabolism

A total of 88 DEGs (13 up-regulated and 75 down-regulated) in the Y2-vs.-Y3 comparison, as well as 14 DEGs (2 up-regulated and 12 down-regulated) in the Y1-vs.-Y3 comparison, were found to be associated with carbon metabolism. In comparison to Y3, most of the carbon metabolism-related genes exhibited up-regulation in both Y1 and Y2. In the Y2-vs.-Y3 comparison, 40 DEGs (6 up-regulated and 34 down-regulated) were involved in glycolysis/gluconeogenesis, while in the Y1-vs.-Y3 comparison, 9 DEGs (1 up-regulated and 8 down-regulated) participated in this pathway. Notably, compared to Y3, a majority of the glycolysis/gluconeogenesis-related genes exhibited up-regulation in both Y1 and Y2. The trend observed in galactose metabolism was consistent with that of carbon metabolism. Specifically, a total of 19 genes (3 up-regulated and 16 down-regulated), along with 10 genes (1 up-regulated and 9 down-regulated) related to galactose metabolism, were found to be significantly up-regulated in both Y1 and Y2 when compared to Y3. These findings suggest that at the 14th day of grain filling, Y3 exhibits significantly weaker carbon metabolism compared to Y1 and Y2.

### 2.8. DEGs Related to MAPK Signaling Pathways

In the Y2-vs.-Y3 comparison, 42 genes (4 up-regulated and 38 down-regulated) were found to be involved in the MAPK signaling pathway, while in the Y1-vs.-Y3 comparison, 10 genes (1 up-regulated and 9 down-regulated) were identified. Notably, these DEGs included BGIOSGA023694 (*EIL2*), BGIOSGA028018 (*MPK2*), BGIOSGA030517 (*Os09g0325700*), BGIOSGA008478 (*EIL1*), BGIOSGA010605 (*SAPK1*), BGIOSGA017659 (*PP2C50*), BGIOSGA007252 (*CATA*), BGIOSGA017630 (*MAPKKK17*), BGIOSGA000957 (*MAPKKK17*), and BGIOSGA023633 (*Cht1*). These findings suggest a significant enhancement of signal transduction-related gene expression in Y1 and Y2, which subsequently mediates downstream processes such as glycolysis progression, carbon metabolism, galactose metabolism, and sucrose starch metabolism.

## 3. Discussion

### 3.1. Positional Variation in Grain Chalkiness Within a Rice Panicle

Chalkiness represents a significant impediment to the improvement of rice quality in China [26]. The metrics for the evaluation of chalkiness include the chalky rice rate, chalkiness area, and chalkiness degree. Prior research has predominantly concentrated on the chalky rice rate, without concurrently addressing the three aforementioned indicators. A holistic examination of these three indices would enhance our understanding of chalkiness formation. Our study revealed that the phenotypic appearance of grains varied depending on their positions within the rice panicle. Specifically, Y1 exhibited the highest chalkiness rate, but had the smallest chalkiness area. Conversely, Y3 demonstrated the lowest chalkiness rate and the largest chalkiness area. The chalkiness rate of Y2 was intermediate between Y1 and Y3, but the chalkiness area was significantly larger than that of Y1 and comparable to Y3. Consequently, Y2 exhibited the highest degree of chalkiness, attributed to the relatively higher chalkiness rate and larger chalkiness area compared to Y1 and Y3. Y1, characterized by early flowering, exhibited the highest chalky rice rate, with its chalkiness degree positioned between Y2 and Y3 due to the minimal chalking area. The positional variations in grain chalkiness within a rice panicle present a complex challenge that cannot be fully understood from a singular perspective. A comprehensive explanation may be achieved by considering factors such as grain size, grain filling patterns, and the deposition of endosperm substances, as elaborated below.

Grain size is a key factor affecting the quality of rice’s appearance. Grain width affects the transport pathway of substances to the endosperm, thereby influencing the supply of essential of essential nutrients required for grain development and ultimately impacting grain quality formation [26]. Previous research has established a strong negative correlation between grain width and grain chalkiness [27]. In this study, Y1 exhibited the smallest grain length-to-width ratio and the greatest grain width, with Y2 and Y3 following in succession. Compared to Y3, the grain filling of Y1 and Y2, which were earlier-flowering spikelets, might have been somewhat impeded due to the increased assimilate transport path caused by the grain width. Consequently, optimizing the grain shape is a key objective in contemporary high-quality rice breeding, as it is anticipated to improve rice’s quality [28].

Significant variations in the grain filling rate are associated with the development of a chalky endosperm in rice. Prior research has demonstrated that a rapid grain filling rate coupled with a shortened duration adversely impacts starch accumulation, thereby increasing chalkiness [29]. In comparison to Y3, both Y2 and Y1 initiated grain filling at an earlier stage and at accelerated rates; however, their filling durations were reduced by 7.10 d and 5.56 d, respectively, relative to Y3. This reduction in duration may have resulted in an insufficient supply of assimilates during grain development, leading to enhanced chalkiness and a decrease in kernel weight. This study presents compelling evidence for variations in endosperm structure across different sections of a panicle. Specifically, in comparison to Y1, the starch granules in the chalky endosperm of Y1 and Y2 exhibited less uniform morphology and a higher prevalence of small starch granules. In agricultural production, it may be necessary to adjust cultivar selection, such as by opting for rice varieties that demonstrate synchronous filling on a panicle, to enhance rice quality and meet market demand.

Starch and protein constitute the primary components of rice endosperm, playing a crucial role in determining its quality [30]. The capacity of amylose and protein accumulation within the rice endosperm are pivotal factors influencing the occurrence of chalkiness [14,15]. Our study on seed storage substances revealed that the amylose and protein contents in Y1 and Y2 were diminished relative to Y3. While no significant difference in protein content was observed between Y1 and Y2, the amylose content in Y1 was significantly lower than that in Y2. Furthermore, the upper grain of the rice panicle exhibited a preferential capacity for assimilate acquisition relative to the medial and basal grains, leading to its enhanced developmental stage. However, the benefits of preferential assimilates allocation to Y1 and Y2 were mitigated by an increased filling rate and greater grain width, which altered the concentration of storage compounds within the endosperm. The results presented above suggest that spikelet positions within the panicle are not definitive determinants of the occurrence of chalkiness. This finding may elucidate the inconsistent results regarding grain chalkiness at various areas within the rice panicle. Instead, chalkiness appears to be influenced by factors such as grain shape, the availability of compounds, and conversion efficiency [11]. The investigation of metabolic processes and genes associated with rice grain chalkiness is necessary, which is connected with the biochemical composition of mature grains.

### 3.2. Changes in Gene Expression and Its Relation to Grain Chalkiness Within a Rice Panicle

Chalkiness in grains represents a multifaceted biological phenomenon influenced by many factors, involving changes in physiological metabolism during grain filling [29]. Elevated temperatures during the grain filling stage accelerate the rate of grain filling, shorten the duration of this period, inhibit starch synthesis, and increase the incidence of chalkiness [14]. Previous research has shown that variations in spikelet positions in the panicle lead to differential capacities of rice components to acquire assimilates [31]. Rice grains in the upper part of the panicle showed the ability to acquire assimilates more preferentially than those in the middle and lower parts of the panicle. In the current study, we conducted an analysis of gene expression in the developing grains across various spikelet positions in a rice panicle. Y3, Y2, and Y1 demonstrated increased expression of genes associated with the MAPK signaling pathway. The Mitogen-Activated Protein Kinase (MAPK) pathway constitutes a highly conserved signaling cascade in eukaryotic organisms, facilitating the transduction of extracellular signals into intracellular response. A complete MAPK cascade comprises MAPK kinase kinase (MAPKKK), MAPK kinase (MAPKK), and MAPK [32]. Upon the reception of external stimuli, MAPKKK initiates the phosphorylation and activation of MAPKK, which in turn catalyzes the phosphorylation and activation of MAPK [33]. Ultimately, the phosphorylated form of MAPK initiates downstream gene activation, thereby modulating subsequent pathways. Prior research has underscored the crucial role of the MAPK pathway in plant growth, development, stress response, and signal transduction [34,35]. A recent study showed that OsMKK3 encoded an MAP kinase kinase that regulated grain size and chalkiness by affecting the cell proliferation of rice spikelet hulls [36]. Overexpression of OsMKK3 in Nip plants increased the grain length and the percentage of grains with chalkiness, which supports our results, indicating the involvement of the MAPK signaling pathway in rice grain filling and seed development.

The flowering sequence in rice typically initiates at the apex of the panicle and progresses incrementally downward [37]. Rice seed development is regulated by the grain-filling process, which is related not only to the flowering time but also to the gene expression of physiological processes [30]. Observations indicate that *Indica* rice exhibits asynchronous grain filling patterns, initiating at the apical portion and subsequently progressing to the middle and lower sections of the panicle. Starch and sucrose metabolism play crucial roles in the grain filling of rice and in determining the grain quality. Transcriptome analysis indicates that the conversion of sucrose to starch is most prominent in developing grains located in the middle section of the rice panicle, with subsequent activity observed in the upper and lower sections. This was supported by subsequent analysis of biochemical components of mature grains and SEM observation of chalky tissues. The above results strongly indicate that the levels of starch and sucrose metabolism affect grain filling and may be involved in the regulation of grain development and chalkiness. This phenomenon may be attributed to substantial temporal variations in the expression levels of genes encoding synthesis enzymes during the grain-filling process, which ultimately result in the formation of chalky endosperms in mature rice grains.

**Figure 6:**
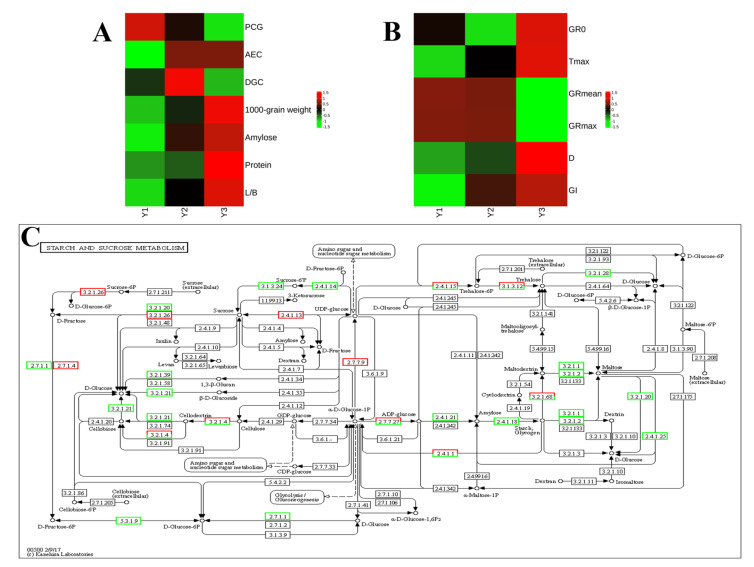
Variation in gene expression among spikelets at different positions within a rice panicle and its relation to grain chalkiness. (**A**) Positional variations in grain chalkiness within a rice panicle. (**B**) Positional variations in grain filling rate within a rice panicle. (**C**) KEGG pathway of starch and sucrose metabolism. Green box indicates down-regulation. Red box indicates up regulation. Y1, the grains attached to the four top primary branches of the rice panicle; Y2, the grains attached to the middle primary branches of the rice panicle; Y3, the grains attached to the four bottom primary branches of the rice panicle; D, active grain filling duration; GR_0_, initial grain filling rate; GR_max_, maximum grain filling rate; GR_mean_, mean grain filling rate; T_max_, times when the growth rate is maximum; GI, grain increment; PCG, percentage of chalky grains; DGC, degree of grain chalkiness; AEC, area of endosperm chalkiness; L/B, grain length/width.

Venn diagram analysis revealed that the starch and sucrose metabolism pathway (ko00500) was enriched across all three comparisons: Y1-vs.-Y2, Y2-vs.-Y3, and Y1-vs.-Y3. The predominant flux in the carbon metabolism of rice grains involves the conversion of sucrose to starch via hexose phosphates [14]. During the grain filling phase, sucrose functions as the principal substrate for starch synthesis and controls endosperm development by modulating the starch content within rice seeds. Sucrose phosphate synthase (SPS) serves as a crucial rate-limiting enzyme in the biosynthesis of sucrose, catalyzing the transformation of UDP-glucose and fructose-6-phosphate into sucrose 6-phosphate and UDP [38]. Additionally, sucrose phosphate phosphatase (SPP) facilitates the hydrolysis of sucrose 6-phosphate, thereby producing sucrose. Hexokinases (HKs) function as rate-limiting enzymes in the glycolytic pathway, initiating glucose metabolism by catalyzing the phosphorylation of glucose to glucose 6-phosphate (G6P), and play an essential role in the sequestration of glucose for subsequent intracellular metabolic processes [39]. In this study, the expression level of hexokinase in Y2 was significantly elevated compared to Y1 and Y3, aligning with the observed trend in glycolytic gene expression. This finding corroborates that the inhibition of starch synthase results in increased rice grain chalkiness [40]. Furthermore, numerous studies have demonstrated the involvement of starch synthase [41], alpha-glucosidase [13], beta-fructofuranosidase [42], and alpha-amylase isozyme [43] in the formation of chalkiness. The metabolism of starch and sucrose is a complex process that influences the development of chalkiness in rice grains and is modulated by the rice genotype. The coordinated expression of genes involved in starch biosynthesis plays a crucial role in producing transparent rice [44]. To gain a more profound understanding of the dynamics of starch and sucrose metabolism genes associated with grain chalkiness, we performed a quantitative analysis of seven genotypes pertinent to starch and sucrose synthesis. Our findings confirmed that Y2 exhibited more significant expression levels of starch and sucrose synthesis genes compared to Y1 and Y3 on the 14th day after flowering. This suggests that this period experienced the most robust activity in the metabolism of sucrose to starch, aligning with the transcriptomic analysis. The majority of these genes were down-regulated, linking metabolic activity to grain filling and contributing to the occurrence of grain chalkiness in the rice panicle (Figure 6).

## 4. Materials and Methods

### 4.1. Plant Material and Growth Conditions

The conventional *Indica* rice variety, Yangdao 6, was cultivated in a micro plot at the experimental farm of the Anhui Academy of Agricultural Sciences (31°86′ N, 117°27′ E) in Hefei City, Anhui Province, China, in 2022. This variety was provided by the Jiangsu Lixiahe Area Agricultural Science Research Institute. Although this variety is widely cultivated in the Yangtze River due to its excellent field yield performance, it exhibited a high production of chalky grains of up to 70%. This quality limitation underscores the imperative for further efforts towards enhancing rice grain appearance.

The seeds were sown on 29 April, and a hill was manually planted with two rice plants spaced 30.0 cm × 13.3 cm on 28 May. In clay loam soil, 225 kg N ha^−1^, 90 kg P_2_O_5_ ha^−1^, and 135 kg K_2_O ha^−1^ were applied to the plants. Rice plants were fertilized with N in a ratio of 5:2:3 (as a basal fertilizer, a top dressing during the tillering stage, and a top dressing during panicle initiation). A single application of P fertilizer was made as a basal fertilizer, while two applications of K fertilizer were made during the basal stage and panicle initiation stage. A floodwater layer of approximately 2–3 cm depth was maintained for a week after transplanting by alternating dry–wet irrigation during the grain-filling stage until natural drying 10 days before harvest on 20 September. Diseases, pests, and weeds were effectively controlled when necessary throughout the rice growing season. During the rice growing season, the average daily temperature was 27.1 °C, the rainfall was 216.0 mm, and the sunshine duration was 923.9 h. The maximum average monthly temperatures occurred in July (35.0 °C) and August (35.2 °C). Elevated temperatures experienced early in seed development can lead to an increase in grain chalkiness.

### 4.2. Sampling

**Figure 7:**
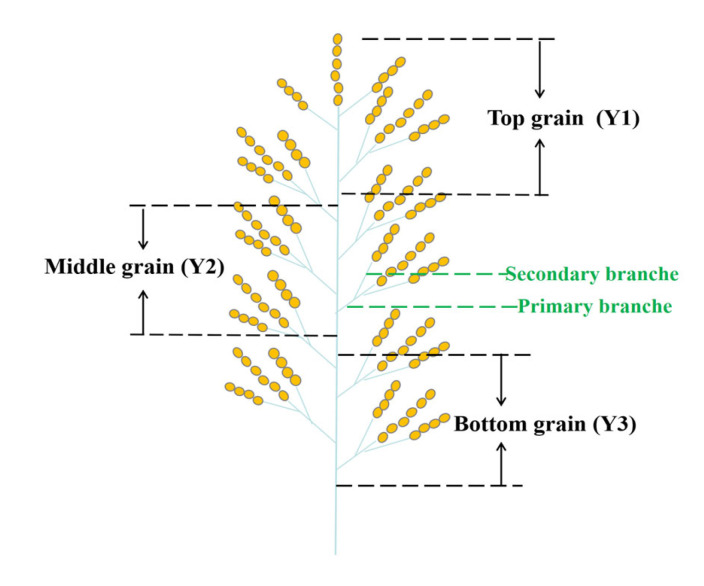
Structure diagram of rice panicle. The grains attached to the four top primary branches are referred to as top grains (Y1), those attached to the four bottom primary branches are known as bottom grains (Y3), while the remaining grains attached to middle primary branches are termed middle grains (Y2).

A total of 300 panicles were marked on the day of flowering. On the 14th day post-flowering, 50 panicles were collected between 9:00 and 9:30 a.m., a period considered critical for seed development due to the occurrence of numerous unique metabolic processes essential for seed formation [13,42]. According to our investigation, the number of primary branches per panicle of Yangdao 6 ranged from 11 to 12, with a predominant occurrence of 12 primary branches per panicle. Grain located in the top, middle, and bottom sections of the rice panicles were categorized into three groups, following the methods of Zhang et al. [11] and Xi et al. [10]. The grains attached to the four top primary branches were referred to as top grains (Y1), those attached to the four bottom primary branches were known as bottom grains (Y3), and the remaining grains attached to middle primary branches were termed middle grains (Y2) (Figure 7). The grain samples from each group were immediately flash-frozen in liquid N and subsequently stored at a temperature of –80 °C for transcriptome analysis. Upon reaching maturity, the remaining tagged panicles were collected, and the grains attached to different spikelet positions of the panicle were categorized into three groups using the same aforementioned method. Three months were spent storing these samples at room temperature after natural air drying, and then they were dehulled to obtain milled rice for the evaluation of grain chalkiness and endosperm structure. Subsequently, the milled rice was ground into powder to facilitate measurements of chemical components.

### 4.3. Phenotype and Physicochemical Properties of the Matured Seeds

The size and chalky characteristics of the milled rice were assessed using a rice appearance quality detection system SC-E (Microtek, Shanghai, China). The area of endosperm chalkiness (AEC) was determined by dividing the degree of grain chalkiness (DGC) by the percentage of chalky grains (PCG) tested. Each sample was measured in triplicate in the present study.

The Scanning Electron Microscopy (SEM) technique was employed to observe the endosperm starch granules in the ventral side of mature rice seeds located at different spikelet positions of the panicle. The grain samples were naturally dried and manually dehulled. Those with a large area of grain chalkiness were selected and gently fractured along the short axis using a razor blade. The resulting cross-sections were mounted on SEM stubs and subsequently sputter-coated with gold under vacuum conditions. Subsequently, using SEM S-3000N (Hitachi, Tokyo, Japan) at a 10 kV accelerating voltage, the starch granule morphology was examined at mag = 500 X, 2.0 KX, and 5.0 KX. A total of 15 high-quality pictures were obtained for each sample. The representative picture was chosen to fully illustrate the ultrastructure characteristics.

Afterwards, the samples were pulverized using an IKA M 20 universal mill (IKA, Guangzhou, China) and utilized for amylose and protein analysis. The amylose content was measured according to Fujita’s method [45]. The nitrogen contents of the flour samples were analyzed using a Smartchem 200 Discrete Auto Analyzer (AMS Alliance, Roma, Italy) after being nitrified in 10 mL H_2_SO_4_ at 350 °C for 2 h. Multiplying the nitrogen content by 5.95 resulted in the protein content. Each sample was measured in triplicate.

### 4.4. Gene Expression Analysis

The trizol reagent kit (Invitrogen, Carlsbad, CA, USA) was used to extract total RNA. The quality of the RNA was evaluated with an Agilent 2100 Bioanalyzer (Agilent Technologies, Palo Alto, CA, USA) and verified by RNase-free agarose gel electrophoresis. cDNA was synthesized from total RNA. An Illumina Nova Seq 6000 platform (Gene Denovo Biotechnology, Guangzhou, China) was used to sequence the resultant cDNA library. Clean reads were generated using Fastp software (version 0.18.0), which facilitated quality control of the raw reads and the exclusion of low-quality data. The procedure for filtering reads is delineated as follows: (1) removal of reads containing adapter sequences; (2) exclusion of reads with more than 10% ambiguous bases (N); (3) elimination of reads composed entirely of adenine (A) bases; and (4) removal of low-quality reads, defined as those in which the proportion of bases with a quality score (Q) of 20 or lower exceeded 50% of the total read length (Table S2). Following the filtering process, we conducted an analysis of the base composition and quality distribution (Table S3) to assess data quality. The Bowtie2 alignment tool (version 2.2.8) was employed to map the remaining reads to the ribosomal RNA database for the removal of rRNA sequences. Gene expression abundance and variations were quantified by calculating FPKM values using RSEM software (http://deweylab.github.io/RSEM/, accessed on 15 August 2024).

To compare two distinct groups, DESeq2 software (https://bioconductor.org/packages/stats/bioc/DESeq2/, accessed on 15 August 2024) was used to analyze the differential expression of RNAs. In this study, transcripts were defined as those with a false discovery rate (FDR) parameter less than 0.05 and an absolute fold change of less than 2-fold. Functional classification of these differentially expressed genes (DEGs) was determined based on Gene Ontology (GO). Additionally, the functions of these DEGs were mapped and enriched in the Kyoto Encyclopedia of Genes and Genomes (KEGG) database. A significance threshold of Q value < 0.05 was applied to identify significantly enriched KEGG pathways.

### 4.5. DNA Database Search and Computer Analysis

To identify members of the starch synthase gene family in rice, nucleotide sequences were searched in the DNA Data Bank of Japan (http://www.ddbj.nig.ac.jp, accessed on 15 August 2024), and Rice genome Annotation Project Database (https://rice.uga.edu/, accessed on 15 August 2024). A multi-sequence alignment of the deduced amino acid sequences was then conducted using the CLUSTAL W program (http://www.genome.ad.jp, accessed on 15 August 2024).

### 4.6. qRT-PCR Analysis

Invitrogen’s SuperScript II (ThermoFisher Scientific, Waltham, MA, USA) was used to reverse-transcribe the isolated RNA samples, with a concentration of five micrograms per sample. Real-time PCR analysis was performed on an aliquot of the first-strand cDNA mixture corresponding to 12.5 ng of total RNA. The amplification program included initial denaturation at 94 °C 5 min, 30 cycles (94 °C 30 s, 55 °C 30 s, and 72 °C 60 s), and then 72 °C 10 min. Primer sequences are shown in Table S1. β-actin served as the internal control gene. The gene expression level was calculated using the 2^−△△Ct^ method. For all test samples and calibration samples, the Ct values of the target genes were normalized with the Ct values of the internal reference gene (β-Actin), and then the ΔCt value of the calibration sample was normalized to the ΔCt value of the test sample. Finally, the relative expression levels of target genes were calculated. Three replicates were conducted for each treatment.

### 4.7. Data Analysis

All of the data in the paper are the results of three biological replicates. Statistical analysis was performed in SPSS 20.0. The differences between the three grain positions related to the physicochemical traits, such as grain chalkiness, grain shape, protein content and parameters of grain filling, were compared using the least significant difference (LSD) test with a significance level of 0.05. All figures were generated in Origin 2022.

## 5. Conclusions

This study analyzed variations in grain filling, gene expression, and grain chalkiness among grains located at different positions within a rice panicle. The PCG in Y1 was the highest, 17.12% and 52.18% higher than Y2 and Y3, respectively. Grains located in the lower part of the panicle exhibited higher amylose and protein contents compared to those situated in the upper or middle sections of the panicle. The increased chalkiness in the rice was characterized by an inadequate accumulation of starch and a loose arrangement of starch granules within the endosperm. Notably, grain filling commenced swiftly in the upper and middle primary branches; however, the duration was 7.10 d and 5.56 d shorter compared to grains from the bottom primary branches. Transcriptomic data revealed that the expression of genes involved in the sucrose-to-starch metabolic pathway was significantly lower in grains from the bottom primary branches compared to those from the top and middle primary branches. This indicates that this metabolic process, which regulates the conversion of sucrose to starch, plays a crucial role in starch synthesis within the developing endosperm and the grain filling, and might be involved in the formation of chalky grains at different positions of the rice panicle.

## Figures and Tables

**Table 1:** Positional variation of grain chalkiness and physicochemical properties within a rice panicle.

Grain Position	PCG	AEC	DGC	1000-Grain Weight(g)	Amylose (%)	Protein (%)	L/B
Y1	73.2 ± 2.68 a	30.1 ± 1.60 b	22.0 ± 0.71 b	28.9 ± 0.02 c	14.56 ± 0.112 b	8.47 ± 0.140 b	2.85 ± 0.012 c
Y2	62.5 ± 1.33 b	41.7 ± 1.06 a	26.1 ± 1.17 a	29.2 ± 0.03 b	15.39 ± 0.514 a	8.53 ± 0.314 b	2.92 ± 0.015 b
Y3	48.1 ± 1.92 c	41.8 ± 0.81 a	20.1 ± 1.18 b	29.7 ± 0.04 a	15.81 ± 0.042 a	9.15 ± 0.406 a	2.99 ± 0.010 a

Note: Y1, the grains attached to the four top primary branches of the rice panicle; Y2, the grains attached to the middle primary branches of the rice panicle; Y3, the grains attached to the four bottom primary branches of the rice panicle; PCG, percentage of chalky grains; DGC, degree of grain chalkiness; AEC, area of endosperm chalkiness; L/B, grain length/width. Values are the means of three replications. Different lowercase letters in the same column indicate significant differences at *p* < 0.05 (n = 3).

**Table 2:** Characteristic parameters of grain filling from different positions within a rice panicle.

Treatment	GR_0_ (mg.grain.d^−1^)	T_max_ (d)	GR_mean_ (mg.grain.d^−1^)	GR_max_ (mg.grain.d^−1^)	D (d)	GI (mg.grain^−1^)
Y1	3.22 ± 0.062 b	3.53 ± 0.103 c	1.48 ± 0.026 a	2.96 ± 0.052 a	14.86 ± 0.054 c	22.00 ± 0.308 c
Y2	2.69 ± 0.479 c	4.75 ± 0.018 b	1.47 ± 0.025 a	2.95 ± 0.050 a	16.40 ± 0.361 b	24.15 ± 0.123 b
Y3	3.68 ± 0.315 a	6.00 ± 0.068 a	1.14 ± 0.018 b	2.28 ± 0.035 b	21.96 ± 0.041 a	24.94 ± 0.340 a

Note: Y1, the grains attached to the four top primary branches of the rice panicle; Y2, the grains attached to the middle primary branches of the rice panicle; Y3, the grains attached to the four bottom primary branches of the rice panicle; D, active grain-filling duration; GR_0_, initial grain-filling rate; GR_max_, maximum grain-filling rate; GR_mean_, mean grain-filling rate; T_max_, times when the growth rate is maximum; GI, grain increment. Different lowercase letters in the same column indicate significant differences at *p* < 0.05 (n = 3).

**Table 3:** List of enriched pathways in Y1-vs.-Y2, Y1-vs.-Y3, and Y2-vs.-Y3.

Enriched Pathway	ID	Y1-vs-Y2	Y2-vs-Y3	Y1-vs-Y3
Metabolic pathways	ko01100	+	+	+
Starch and sucrose metabolism	ko00500	+	+	+
Carbon fixation in photosynthetic organisms	ko00710	+	+	
Carbon metabolism	ko01200		+	+
Glycolysis/gluconeogenesis	ko00010		+	+
Galactose metabolism	ko00052		+	+
MAPK signaling pathway–plant	ko04016		+	+
Diterpenoid biosynthesis	ko00904	+		
Carotenoid biosynthesis	ko00906	+		
Protein processing in endoplasmic reticulum	ko04141	+		
Biosynthesis of secondary metabolites	ko01110	+		
Amino sugar and nucleotide sugar metabolism	ko00520	+		
Aflatoxin biosynthesis	ko00254	+		
Riboflavin metabolism	ko00740	+		
Terpenoid backbone biosynthesis	ko00900		+	
Pyruvate metabolism	ko00620		+	
Ascorbate and aldarate metabolism	ko00053		+	
Glucosinolate biosynthesis	ko00966			+
Alanine, aspartate, and glutamate metabolism	ko00250			+
Neomycin, kanamycin, and gentamicin biosynthesis	ko00524			+
Butanoate metabolism	ko00650			+

Note: Y1, Y2, and Y3 indicate the grains attached to the four top primary branches, the remaining middle primary branches, and the four bottom primary branches of the rice panicle, respectively. “+” indicates that the process participates in the path.

## Data Availability

The data presented in this study are available in the Supplementary Files.

## Supplementary Materials

The following supporting information can be downloaded at: https://www.mdpi.com/article/10.3390/plants14020244/s1.
