# Supplementary material for: Molecular Mechanisms of Grain Chalkiness Variation in Rice Panicles
Source: Plants (Basel). 2025 Jan 16;14(2):244. doi: 10.3390/plants14020244 (PMC11768284; doi:10.3390/plants14020244)
Supplement: Supplementary file 1 [file plants-14-00244-s001.zip › plants-3325917-supplementary/supplement/supplement.pdf]

Table S1: Primers used for qRT-PCR

Gene	Forward sequence（5’-3’）	Reverse sequence（5’-3’）
sucrose-phosphate synthase (SPS1)	AAAGGGTGATCTGAAGGCCG	CACAATGGCCACTGCCAATC
hexokinase-8-like (HXK8)	TCTGCGATTCAAGGCTGACC	GCATTGCAGAAATGTGCGGA
hexokinase-9-like (HXK9)	CCCTCCGGGGGTGAGAAAG	TTCGACGTTCTTTGCCTCCTAA
alpha-glucosidase (Os06g0675700)	CGTCATCATTGACCCAGGGA	ATGTCCTCCTTGATCGCTCG
beta-fructofuranosidase, insoluble isoenzyme 7-like (CIN7)	CCACCTCTTCCTTCTTGCGT	TGGTACATAGGCCCATTCGG
alpha-amylase isozyme 3A-like (AMY1.2)	GAAGCAGGGTGGTTGGTACA	TCCCGGCATGTAGCCTTGAG
β-Actin	GACATTCAGCGTTCCAAGCCATGTAT	TGGAGCTTCCATGCCGATGAGAGAA

Table S2 Transcriptome data filtering statistics table

Sample	RawDatas	CleanData(%)	Adapter(%)	LowQuality(%)	polyA(%)	N(%)
Y1-1	36306744	36028732 (99.23%)	16996 (0.05%)	260750 (0.72%)	0 (0.00%)	266 (0.00%)
Y1-2	44282576	43967718 (99.29%)	18388 (0.04%)	296470 (0.67%)	0 (0.00%)	0 (0.00%)
Y1-3	40494424	40188216 (99.24%)	15038 (0.04%)	291122 (0.72%)	0 (0.00%)	48 (0.00%)
Y2-1	59707300	59239750 (99.22%)	34628 (0.06%)	431256 (0.72%)	0 (0.00%)	1666 (0.00%)
Y2-2	40798156	40505602 (99.28%)	19852 (0.05%)	272392 (0.67%)	0 (0.00%)	310 (0.00%)
Y2-3	39512486	39225754 (99.27%)	15806 (0.04%)	270840 (0.69%)	0 (0.00%)	86 (0.00%)
Y3-1	42618372	42302252 (99.26%)	16858 (0.04%)	299262 (0.70%)	0 (0.00%)	0 (0.00%)
Y3-2	39762930	39444512 (99.20%)	19740 (0.05%)	298522 (0.75%)	0 (0.00%)	156 (0.00%)
Y3-3	41497666	41158262 (99.18%)	14088 (0.03%)	325176 (0.78%)	0 (0.00%)	140 (0.00%)

Table S3 Transcriptome base information statistics table

Sample	RawData (bp)	BF_Q20(%)	BF_Q30(%)	BF_N(%)	BF_GC(%)	CleanData(bp)	AF_Q20(%)	AF_Q30(%)	AF_N(%)	AF_GC(%)
Y1-1	5446011600	5298434748 (97.29%)	5040770227 (92.56%)	53893 (0.00%)	2781799851 (51.08%)	5379997559	5248826046 (97.56%)	4997073337 (92.88%)	48554 (0.00%)	2746458302 (51.05%)
Y1-2	6642386400	6462133755 (97.29%)	6144140540 (92.50%)	23183 (0.00%)	3391139786 (51.05%)	6567645919	6407078892 (97.56%)	6095867618 (92.82%)	22934 (0.00%)	3350711158 (51.02%)
Y1-3	6074163600	5886184542 (96.91%)	5580450183 (91.87%)	24219 (0.00%)	3147582538 (51.82%)	6002143214	5832878698 (97.18%)	5534309059 (92.21%)	22660 (0.00%)	3108079318 (51.78%)
Y2-1	8956095000	8660794258 (96.70%)	8173965056 (91.27%)	263652 (0.00%)	4692910009 (52.40%)	8849128338	8582043225 (96.98%)	8105304974 (91.59%)	219617 (0.00%)	4633506694 (52.36%)
Y2-2	6119723400	5952856273 (97.27%)	5659409471 (92.48%)	61340 (0.00%)	3174179274 (51.87%)	6044002847	5895131543 (97.54%)	5608413761 (92.79%)	54923 (0.00%)	3133105012 (51.84%)
Y2-3	5926872900	5772818224 (97.40%)	5509210117 (92.95%)	25259 (0.00%)	3094471824 (52.21%)	5854443240	5718324946 (97.67%)	5461420858 (93.29%)	22766 (0.00%)	3054466738 (52.17%)
Y3-1	6392755800	6219809842 (97.29%)	5913596456 (92.50%)	22239 (0.00%)	3197079844 (50.01%)	6316150979	6163196697 (97.58%)	5863726261 (92.84%)	21982 (0.00%)	3156217382 (49.97%)
Y3-2	5964439500	5789350732 (97.06%)	5491495412 (92.07%)	60544 (0.00%)	2987434816 (50.09%)	5888428200	5732795653 (97.36%)	5441853360 (92.42%)	56561 (0.00%)	2946872584 (50.05%)
Y3-3	6224649900	6036729342 (96.98%)	5732421911 (92.09%)	35507 (0.00%)	3130312445 (50.29%)	6141770783	5975133416 (97.29%)	5678783498 (92.46%)	33226 (0.00%)	3086036260 (50.25%)
